# Age Differences in the Tradeoff between Proactive and Reactive Cognitive Control in Emotional Information Processing

**DOI:** 10.3390/brainsci12081043

**Published:** 2022-08-05

**Authors:** Ni Zhang, Jingxin Wang

**Affiliations:** 1 Faculty of Psychology, Tianjin Normal University, Tianjin 300387, China; 2 Center of College Student Mental Health, Weifang Medical University, Weifang 261053, China; 3 Academy of Psychology and Behavior, Faculty of Psychology, Tianjin Normal University, Tianjin 300074, China

**Keywords:** proactive control, reactive control, emotional information processing, aging

## Abstract

Greater well-being in older adults stems from more effective emotion regulation strategies, highlighting the role of cognitive control. However, cognitive control involves different subsystems, and it is still unclear whether different subsystems have different effects on different emotional information processing. The Dual Mechanisms of Control (DMC) theory postulates that cognitive control can operate in two distinct modes, namely proactive control (a “proactive” preparatory mode) and reactive control (a “reactive” wait-and-see mode). This study created an emotional AX-CPT task to explore differences in cognitive control tradeoff between youth and older adults when processing emotional information. The results found that youth had significantly higher error rates on the emotional-neutral sequence than older adults regardless of the valence of emotional information; only in the negative condition did older adults have higher error rates on both the sad-sad and neutral-sad sequences than youth; this phenomenon was not found in the positive condition. The study showed that, in emotional information processing, youth preferred proactive control over older adults; in negative information processing, older adults preferred reactive control strategies over youth; in positive information processing, older adults showed a similar cognitive control pattern to youth, and proactive control was enhanced.

## 1. Introduction

People become happier with age and show a “positivity effect” on emotional processing, i.e., a greater focus on positive rather than negative stimuli [1,2]. The reason for this phenomenon may be related to the fact that older adults regulate emotions more effectively [3,4]. For this reason, age differences in cognitive processes, and their effects on emotion regulation, have become a hot topic of research. Cognitive strategies can focus on positive information and suppress negative information to enhance emotional well-being, resulting in “positivity effects” on attention and memory [5].

Socioemotional Selectivity Theory (SST) is a lifespan development theory related to motivation [6]. From the perspective of motivation, the theory suggested that the elderly show positivity effects in emotional information processing in order to achieve the emotional goal of improving and maintaining their own positive emotional experience, thus preferentially allocating limited cognitive resources to the processing of emotional information, especially positive information [1,7,8]. Selective cognitive processing is a component of effective emotion regulation [9], and the cognitive control hypothesis suggested that prioritizing the achievement of emotional goals in older adults required cognitive effort. Therefore, only the elderly with high cognitive control function or adequate cognitive resources will show stronger positivity effects [10], which emphasized that cognitive control played a key role.

Cognitive control refers to the psychological process in which individuals flexibly adjust and control their cognition and behavior by inhibiting inappropriate behavior impulses according to current task objectives [11,12]. This process directly determines the individual’s thoughts and behaviors, especially when the individual is faced with the task situation of interference or conflict. In order to achieve the final task, cognitive control plays a crucial role. Cognitive control allows individuals to allocate attention and memory in various ways to satisfy emotional needs [13]. Previous studies on cognitive processing have confirmed that aging leads to cognitive decline in the elderly [14,15,16]. Inhibition deficit theory and goal maintenance theory have proposed a direct mechanism of age-related cognitive control deficit [17,18].

Empirical studies have been conducted to examine the role of cognitive control as an integrated function more in terms of the level of executive function, or the number of cognitive resources, in emotional information processing in youth and older adults. For example, the positive effects on situational memory or attention in older adults disappear when attention is distracted [19,20,21]. Older adults who can exercise more flexible top-down control under a distraction task pay more attention to positive information [3]. Yet cognitive control involves different subsystems [22]. Braver proposed that Dual Mechanisms of Cognitive Control Account (DMC) divides cognitive control into proactive control and reactive control, which provides a new perspective for understanding cognitive control [23]. Proactive control is similar to the above definition of cognitive control. It refers to a cue-driven control in which the individual forms a corresponding response preparation to complete the cognitive task according to the cue information before the response and is greatly influenced by top-down information processing [24]. Proactive control prevents conflict by maintaining the representation of cue information before response, which is an early selection process and can control behavior more effectively. However, it relies on reliable cue information and requires more cognitive and physiological resources to maintain the continuous representation of cue information. Reactive control refers to the probe-driven control that individuals who complete cognitive tasks by reactivating task-related cues when they are about to respond [24], which is greatly influenced by external information input. Reactivity control is a kind of late modification processing, in which there is less information representation, less reaction-related preparation, and not too much cognitive load. Compared with proactive control, it has a better adaptability and lower resource consumption, but its control effect is not ideal. Thus, individuals are free to choose one, or weigh between the two modalities according to the current task requirements, adjusting their weights to form the most beneficial control mode for the task [25].

Previous studies have mainly examined the effects of reward and emotional state on cognitive control performance in youth [26,27,28,29]. It is found that reward motivation leads individuals to prefer proactive control [26,28,30]. A recent fNIRS study found that individuals invested more cognitive resources to represent cue information in the cue stage under the condition of expected punishment, which made individuals prefer proactive control strategies [31]. There are still no consistent conclusions about the influence of emotion on cognitive control. For example, some studies have found that a negative emotional state causes damage to proactive control and reactive control [32,33], while some researchers found that a negative emotional state can improve the performance of task switching [34]. Positive emotions can reduce an individual’s proactive control but have little influence on reactive control [35]. However, some researchers found that positive emotions enhanced the flexibility of working memory representation, and reactive control was improved, while proactive control was not affected [36]. In addition, the natural decline in prefrontal function in older adults impaired the ability to maintain representations, thus shifting individuals from proactive to reactive control as they age [37,38,39].

Emotion regulation goals dominate in old age. Therefore, it is reasonable to speculate that older adults’ performance of cognitive control tradeoff in the processing of different emotional information is likely to be different from that of youth. This study focuses on changes in cognitive control tradeoff in older adults when processing emotional information in the face of different emotional information and how they differ compared to the youth.

The AX-continuous Performance Task (AX-CPT) is a classical paradigm to investigate proactive control and reactive control [24,40,41]. The task was composed of cue stimuli and probe stimuli, with English capital letters as materials. The cue stimuli were divided into target stimulus A and non-target stimulus B (B stood for any letter except A and probe stimulus), and the probe stimuli were divided into target stimulus X and non-target stimulus Y (Y stood for any letter except X and cue stimulus). This results in four sequence types: AX, AY, BX, and BY. The subjects were required to make the only target response quickly and accurately to the detection stimulus X (AX sequence) after the cue stimulus A, while the other sequences (including AY sequence, BX sequence, and BY sequence) were all non-target responses. Since the AX trials accounted for 70% of the total trials, and the other three trials accounted for 10% each, subjects had a strong tendency to respond to the target stimulus (A or X) [42]. According to dual cognitive control theory, proactive control enhances the representation of cues, which leads to an increase in the error rate of the AY sequence or a decrease in the error rate of the BX sequence. Reactive control can lead to an increase in the error rate of BX or a decrease in the error rate of AY [24]. Therefore, the tradeoff between proactive and reactive control can be examined by analyzing behavior on the AY or BX sequences [28,42,43]. To this end, the present study improved the classical AX-CPT by replacing letters on cue stimuli and probe stimuli with emotional faces in a first attempt to examine the differences in cognitive control tradeoff between youth and older adults in the processing of different emotional information.

According to previous research, youth were more inclined to process negative emotional information [44], whereas older adults were more inclined to attend to, and remember positive rather than negative information [45,46]. Therefore, it is hypothesized that, in the positive information condition, older adults are more likely to intrinsically represent positive emotional cue information and thus will be more inclined to use proactive control strategies compared to youth, whereas in the negative information condition, older adults are likely to have difficulty in consistently representing cues due to the negative attention bias of youth and more avoidance of negative information, and thus are likely to be more inclined to use reactive control strategies. In contrast, the proactive control bias of youth would not be influenced by the emotional validity of the information.

## 2. Methods

### 2.1. Participants

According to previous findings [38,47], the calculations of G*Power 3.1 [48,49], for the between-subjects repeated measures ANOVA applied in this study. The total sample size predicted a level of statistical power of 80% at a significance level of 0.01 with a medium effect size (0.25) [50] was at least 22 subjects. Thirty-two college students and 26 older adults over 60 years of age were randomly recruited for this study.

Eligibility criteria included: (a) no history of neurological issues (e.g., stroke, dementia, major head injury); (b) no history of uncontrolled medical conditions (e.g., diabetes, cardiovascular diseases); (c) no current diagnoses of mood disorders (e.g., depression or anxiety disorders) or self-reported “extremely severe” depression/anxiety symptoms with the Depression, Anxiety, and Stress Scale (DASS-21) [51]; (d) all older adults participating in the experiment had at least 12 years of education; (e) all subjects had normal or corrected visual acuity, were right-handed, and had never participated in similar experiments before. One of the young subjects failed to record the experimental data properly, and one young person voluntarily asked to withdraw during the experiment. Young adults were recruited from an undergraduate participant pool and older adults were recruited from the local community. Subjects who participated in the experiment signed an informed consent form for the experiment and received a certain amount of money as an honorarium for their participation. Finally, 30 young adults (12 males, 18 females, mean age 19.53 years) and 26 older adults (15 males, 11 females, mean age 69.65) were included in the follow-up data analysis. The study was approved by the Ethics Committee of the faculty of psychology, Tianjin Normal University.

Independent sample *t*-tests were performed using IBM SPSS 25.0 software for demographic characteristics of young and older adults, as well as on scores on the FTP, PANAS, DASS-21, and MoCA scales (see Table 1). The results showed that the perception of future time was significantly shorter in older adults (*M* = 25.92, *SD* = 5.92) than in youth (*M* = 38.43, *SD* = 4.72), *t* = 8.80, *p* < 0.001, and Cohen’s *d* = 2.36, indicating that older adults significantly perceived the limited nature of future time compared to youth; There was no significant difference between the two groups in the scores of positive-negative emotion rating and depression-anxiety-stress scales (*p* > 0.05), indicating that the emotional states of all subjects were within the normal range; in the MoCA scores, all subjects were above 20, and the scores of the youth group (*M* = 26.87, *SD* = 1.41) were significantly higher than those of the older group (*M* = 25.65, *SD* = 2.56), *t* = 2.15, *p* < 0.05, Cohen’s *d* = 0.60, indicating that all subjects were within the normal range of cognitive function and that the youth group had a better cognitive function.

### 2.2. Instrument and Experimental Materials

*The Future Time Perspective* (FTP) scale was developed by Lang and Carstensen to measure the future time perception of older adults [52]. Fung translated the questionnaire into Chinese and conducted a localized study on the future time perception of Chinese older adults and revised the way of calculating the statistical scores of the questionnaire, changing the original t-score to the mean statistical method [53]. In this study, the revised version of the FTP scale was used, and the mean was used as the statistical indicator in the analysis of the results, with higher scores indicating more perceived future time.

*Positive Affect and Negative Affect Scale* (PANAS) was developed by Watson and Clark to assess individuals’ positive and negative emotions [54]. The Chinese version of the Positive Affect and Negative Affect Scale was revised by Huang et al. and has been shown to have good reliability in both community-based and urban elderly populations [55,56]. The scale contains two factors, positive and negative emotions, consisting of 10 adjectives describing positive emotions and 10 adjectives describing negative emotions, respectively. Each adjective is scored on a five-point scale, ranging from 1 = “almost none” to 5 = “extremely many”, with higher scores indicating stronger positive or negative emotions.

*The Depression-Anxiety-Stress Scale (DASS-21)* was developed by Lovibond et al. and consists of three subscales with 42 items [51]. The DASS-21 is a revised and streamlined version of the DASS, which retains seven items for each of the three subscales of depression, anxiety, and stress, while keeping the dimensions of the original scale unchanged, to improve the efficiency of identifying and assessing the symptoms of the corresponding mood disorders. A four-point scale was used, ranging from 0 = “not at all” to 3 = “fully”, with higher scores indicating more intense negative emotional experiences. Gong et al. reported internal consistency of 0.77, 0.79, and 0.76 for the depression, anxiety, and stress subscales, respectively; the total scale had an internal consistency coefficient of 0.89 [57].

*The Montreal Cognitive Assessment* (MoCA) was developed by Nasreddine et al. in Canada based on clinical experience and concerning the cognitive items and scores of the MMSE (Brief Mental State Examination) and includes visual spatial function (5 points), naming (3 points), attention (6 points), repetition of sentences (2 points), fluency (1 point), abstraction (2 points), delayed recall (5 points), and orientation (6 points), for a total score of 30 points, plus 1 point for the total score if the subject has ≤12 years of education [58]. Lower scores indicated a more severe impairment of cognitive function. Subjects with a total MoCA score ≥ 20 were included in the final tally of this study [59].

*Emotional AX-CPT task* Since emotional faces have more socio-emotional significance compared to other emotional materials, the experiments were conducted by replacing the letter materials with emotional faces based on retaining the logic of the classical AX-CPT experiment, with the cue stimuli being emotional faces (divided into happy or sad faces) or neutral faces, and the probe stimuli also being emotional faces (divided into happy or sad faces) or neutral faces, resulting in four sequence types under two emotions: an emotion-emotion (happy-happy or sad-sad) sequence, an emotion-neutral (happy-neutral or sad-neutral) sequence, a neutral-emotion (neutral-happy or neutral-sad) sequence, and a neutral-neutral sequence. Subjects were required to make a unique target response to the probe stimulus that appeared after the cued stimulus—a certain type of emotional face that was also that type of emotional face (i.e., emotional-emotional sequence)— both quickly and accurately, and the other sequences (emotional-neutral sequence, neutral-emotional sequence, neutral-neutral sequence) made non-target responses. In the experiment, emotion-emotion trials accounted for 70% of the total trials, and the other three trials each accounted for 10% (see Table 2). The behavioral performance on the emotion-neutral or neutral-emotion sequences was analyzed as a way to examine the trade-off between subjects’ proactive and reactive control in emotional information processing.

The present study used 36 emotional faces of 6 elderly (3 males, 3 females) and 6 youth (3 males, 3 females) from the Faces of Emotion System (FACES) [60]: 12 images each of pleasant, sad, and neutral faces. Twenty Chinese youth (7 males, 13 females, mean age 20.35 years, age range 19–22 years) and 20 Chinese elderly (9 males, 11 females, mean age 70.05 years, age range 62–82) were selected to evaluate the selected emotional faces, and the results are shown in Table 3.

The level of agreement with the three types of emotional faces: pleasant, neutral, and sad ranged from 92.08% to 99.58%. ANOVA on arousal showed that the main effect between groups (youth and elderly groups) was not significant (*p* > 0.05) and the main effect of emotional type was significant (*p* < 0.001, *η*^2^ = 0.84). The results of Bonferroni post hoc multiple comparisons showed that arousal was significantly higher for both pleasant and sad faces than for neutral faces (*p* < 0.001), while for pleasant and sad faces arousal was not significantly different between pleasant and sad faces (*p* > 0.05). The above results suggest that the selected foreign emotional face pictures are equally applicable to Chinese youth and the elderly.

### 2.3. Experimental Design

A mixed design of 2 (Age: young vs. older) × 2 (Emotional faces: happy vs. sad) × 4 (Sequence type: emotional-emotional/emotional-neutral/neutral-emotional/neutral-neutral) was used, with the age as a between-subjects variable, emotional faces, and sequence type as within-subjects variables, and dependent variables of error rate and reaction time of correct responses.

### 2.4. Experimental Procedure

**Figure 1:**
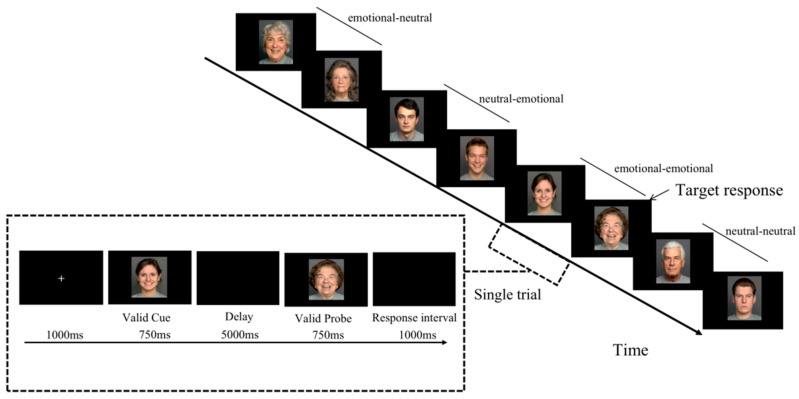
Experimental flow diagram (the left diagram shows the flow diagram of the single-trial experiment; the right diagram shows the flow diagram of the emotional AX-CPT task and its stimulus ratio).

All subjects completed the emotional AX-CPT task individually, and the specific experimental procedure is shown in Figure 1. Subjects were asked to press the “J” key with the right index finger for target responses when the “emotional-emotional” sequence appears and press the “F” key with the left index finger for non-target responses when the other face sequences (emotional-neutral/neutral-emotional/neutral-neutral) appear. The left- and right-handed key responses were counterbalanced between subjects. Cue faces and probe faces were balanced for gender and age, and cue- and probe- faces were not repeatedly presented before and after. The experiment was divided into a practice experiment and a formal experiment, and the practice experiment consisted of nine trials to familiarize the subjects with the experimental procedure, with a correct rate of 80% or more. Each block presented subjects with only one type of emotional stimuli (i.e., sad or happy) and contained 100 trials, with the order of presentation of the two types of emotional materials being balanced among subjects and the four trial types being presented randomly within the blocks. The full experimental task took approximately 40 min, and a certain amount of money was given at the end of the experiment. E-prime 2.0 was used to write, present, and summarize the data. IBM SPSS 25.0 was used for statistical analysis.

## 3. Results

### 3.1. Error Rate

**Figure 2:**
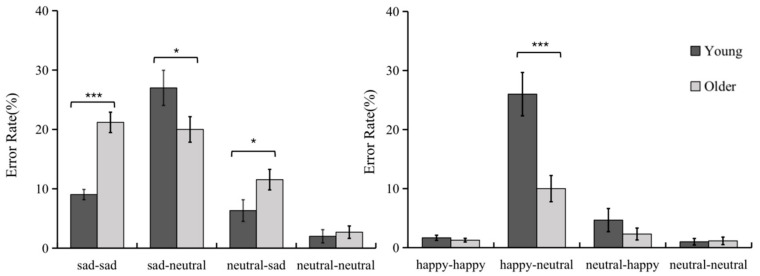
Error rate performance of youth and older adults completing the emotional AX-CPT task. * *p* < 0.05; *** *p* < 0.001.

A 2 (Age: young vs. older) × 2 (Emotional faces: happy vs. sad) × 4 (Sequence type: emotional-emotional/emotional-neutral/neutral-emotional/neutral-neutral) repeated measures ANOVA was conducted on error rates (see Figure 2). Results revealed a significant main effect of emotional faces, *F*(1, 54) = 74.38, *p* < 0.001, *η*^2^ = 0.58, with a significantly higher error rate in the sad condition (12.50 ± 5.24%) than in the happy condition (6.00 ± 5.99%), *p* < 0.001; a significant main effect of sequence type, *F*(3, 162) = 69.69, *p* < 0.001, *η*^2^ = 0.56, post hoc comparisons revealed that the error rate on the emotional-neutral sequence (20.70 ± 12.72%) was significantly higher than on the emotional-emotional (8.30 ± 3.74%) and neutral-emotional sequences (6.20 ± 6.73%), and the error rate on the emotional-emotional and neutral-emotional sequences was significantly higher than on the neutral-neutral sequence (1.70 ± 3.74%). The interaction between age and emotional faces was significant, *F*(1, 54) = 24.45, *p* < 0.001, *η*^2^ = 0.31, and simple effects analysis revealed significant differences in error rates between youth and older adults for both happy and sad conditions. The interaction between age and sequence types was significant, *F*(3, 162) = 14.47, *p* < 0.001, *η*^2^ = 0.21. Simple effects analysis found significant differences in error rates between young and older on the emotional-emotional and emotional-neutral sequences, and non-significant differences in the other sequences. The interaction between emotional faces and sequence types was significant, *F*(3, 162) = 12.32, *p* < 0.001, *η*^2^ = 0.19; simple effects analysis found significant differences in error rates across emotional conditions for all three sequences, except for non-significant differences in error rates for neutral-neutral sequences. In addition, the triple interaction of group, emotional faces, and sequence types was significant, *F*(3, 162) = 12.32, *p* < 0.05, *η*^2^ = 0.051. Further simple effects analysis revealed that on the sad-sad and neutral-sad sequences, the error rate was significantly higher in the older group than in the youth (*p* < 0.001; *p* < 0.05); on the sad-neutral sequence, the youth group had an error rate higher than in the older group (*p* = 0.06). Subjects’ error rates in completing the AX-CPT task in the happy condition were significantly higher in youth than in older adults only on the happy-neutral sequence (*p* < 0.001). The above results indicate that, in the processing of different emotional information, youth were more inclined to proactive control than older age; in the processing of negative information, older age was more inclined to reactive control strategies than youth; in the processing of positive information, older age showed similar cognitive control patterns as youth, and youth had more proactive control.

### 3.2. RT

**Figure 3:**
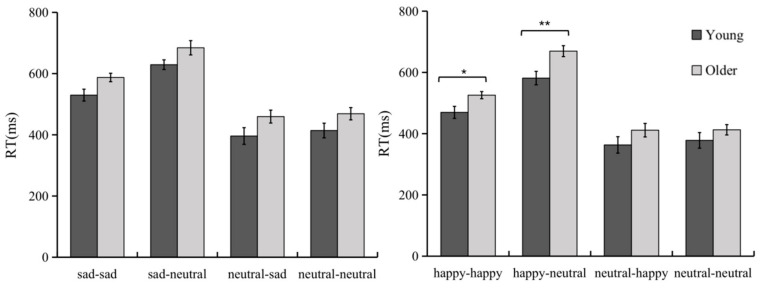
Reaction-time performance of youth and older adults completing the Emotional Faces AX-CPT task. * *p* < 0.05; ** *p* < 0.01.

A 2 (Age: young vs. older) × 2 (Emotional faces: happy vs. sad) × 4 (Sequence type: emotional-emotional/emotional-neutral/neutral-emotional/neutral-neutral) repeated measures ANOVA was conducted on reaction time (see Figure 3). The results revealed a significant main effect of age, *F*(1, 54) = 5.12, *p* < 0.05, *η*^2^ = 0.09, with a longer mean reaction time in the older group than in the younger group; the main effect of emotional faces was significant, *F*(1, 54) = 31.72, *p* < 0.001, *η*^2^ = 0.37, with the mean reaction time in sad faces compared to happy faces (476.46 ± 102.30) (521.12 ± 96.01) being longer; the main effect of sequence type was significant, *F*(3, 162) = 273.94, *p* < 0.001, *η*^2^ = 0.84, and the reaction time on the emotional-neutral sequence was significantly longer than the other three sequences; the reaction time on the emotional- emotional sequence was significantly longer than the neutral-emotional and neutral-neutral sequences. None of the interactions were significant (*p* > 0.05).

A 2 (Age: young vs. older) × 4 (Sequence type: emotional-emotional/emotional-neutral/neutral-emotional/neutral-neutral) repeated measures ANOVA was conducted on reaction times in the sad condition and the happy condition, respectively. Results revealed a significant main effect of age under the sad condition, *F*(1, 54) = 5.07, *p* < 0.05, *η*^2^ = 0.09; a significant main effect of sequence type, *F*(1, 54) = 142.07, *p* < 0.001, *η*^2^ = 0.73; the interaction between the two was not significant (*p* > 0.05). Under the happy faces, the main effect of age was significant, *F*(1, 54) = 4.30, *p* < 0.05, *η*^2^ = 0.07; the main effect of sequence type was significant, *F*(3, 162) = 226.19, *p* < 0.001, *η*^2^ = 0.81; the interaction between the two was borderline significant, *F*(3, 162) = 2.36, *p* = 0.07, *η*^2^ = 0.04, and the simple effect analysis revealed that the reaction time was significantly longer in the older group than in the younger group on both happy-happy and happy-neutral sequences, and the differences between the other two sequences were not significant. The above results suggest that there is a general slowing down of response times in older adults compared to youths under different sequence tasks; older adults showed longer response times on the happy-neutral sequence, indicating that older adults put more cognitive effort into actively forming representations of the happy cues.

### 3.3. Proactive Control Index (PBI)

Referring to previous studies [22,61], we measured the relative propensity of individuals to adopt proactive control by calculating the proactive control index (PBI) to further examine the cognitive control tradeoff between youth and older adults in emotional information processing. In this paper, PBI = (emotional-neutral sequence − neutral-emotional sequence)/(emotional-neutral sequence + neutral-emotional sequence) and its value ranges from −1 to 1, with a larger index indicating a greater tendency to adopt proactive control [22,30,62].

A 2 (Age: young vs. older) × 2 (Emotional faces: sad vs. happy) repeated measures ANOVA was performed on the PBI index calculated from the error rate. Results showed a significant group main effect, *F*(1, 54) = 6.42, *p* < 0.05, *η*^2^ = 0.11, and a PBI index closer to 1 for youth, indicating a greater tendency for proactive control in youth.

To examine the possible effects of baseline group differences, correlation analyses were performed between relevant demographic or cognitive variables and PBI scores. The results showed that, in older adults, RT PBI scores in the sad condition were negatively correlated with NA scores (*r* = −0.437, *p* = 0.026), i.e., the higher the subjects’ negative emotion, the lower the active control index in response to the sad condition. In contrast, among youth, RT PBI scores in the sad condition were positively correlated with NA scores (*r* = 0.405, *p* = 0.026), i.e., the higher the subjects’ negative emotion score, the higher the proactive control index in response to the sad condition. This interesting finding was not found in the happy condition.

## 4. Discussion

The present study was the first attempt to examine age differences in cognitive control tradeoff in emotional information processing by manipulating emotional potency, and stimulus sequence type using the emotional AX-CPT paradigm. The findings revealed that youth had significantly higher error rates than older adults on sad-neutral and happy-neutral sequences, i.e., youth were more inclined to employ proactive control strategies regardless of the type of emotional information processing, which is in line with the results of previous studies [39,47]. Proactive control is a cue-driven control, and because proactive control requires individuals to continuously represent cue information, it requires significant consumption of cognitive resources. In contrast, youth have abundant cognitive resources relative to older adults, and thus are more likely to exhibit proactive control tendency in cognitive processing.

A more interesting finding is that there are some differences in the cognitive control tradeoff between youth and older adults in the processing of different emotional information. Specifically, in the negative condition, older adults had significantly higher error rates on both the sad-sad and neutral-sad sequences than youth, i.e., older adults were more inclined to adopt reactive control strategies than youth in the processing of negative information. This is similar to the finding in the classical AX-CPT task that older adults tend to adopt reactive control strategies more often as they age [63,64]. Reactive control is a probe-driven control that requires less cognitive resources because reactive control does not require individuals to continuously represent cue information. In addition, the authors suggest that older adults’ greater tendency to use reactive control strategies in negative conditions may also be related to the fact that it is more difficult for older adults to cognitively process grief information and attempt to form target response tendency than youth. This is further supported by the fact that the error rate on the sad-sad sequence is significantly higher in older adults than in youth. Thus, difficulties in processing sad information make it more difficult for older adults to form internal representations of sadness cues to prevent conflict, which in turn increases their error rate on the neutral-sad sequence.

In the happy condition, older adults showed a similar pattern of cognitive control to youth, i.e., the elderly’s error rate under happy-neutral sequence was higher than the other sequences, suggesting that older adults enhanced the characterization of happy cues, thus, the error rate under the happy-neutral sequences rose while the error rate under the neutral-happy sequence decreased. In addition, older adults showed longer response times than youth on the happy-neutral sequence, which further indicates that older adults exerted more cognitive effort to actively form representations of pleasant cues. The results also supported Socioemotional Selectivity Theory’s explanation of the positivity effect, namely that older adults’ preferential processing of positive information is controlled processing [2,10,46,65], rather than automated processing, due to cognitive decline [66].

In addition, older adults generally have longer reaction times than younger adults in the processing of different emotional information, a result that is consistent with the generalized slowing hypothesis, which states that processing slows down with healthy aging [67,68]. At the same time, older adults did not show a cognitive control pattern similar to the error rate, i.e., in the emotional-neutral condition, the youth had significantly higher error rates than older adults, yet this pattern was not replicated in response times, but rather showed a pattern of generally higher response times for older adults than youth, which may reflect a tradeoff between speed and accuracy in which older adults forgo fast responses in order to ensure higher accuracy.

## 5. Limitations and Future Directions

The present study yielded some interesting behavioral findings. It further enriches the explanation of the role of cognitive control in the positive effects of aging. However, there are several shortcomings: first, this study did not distinguish between emotional arousal of emotional faces. Low arousal and high arousal emotional information involve different processing mechanisms. High arousal stimuli primarily trigger automatic attentional capture to ensure that information is processed preferentially; increasing the processing of low arousal stimuli involves top-down cognitive processes and therefore requires the involvement of cognitive resources. Automatic processing of high-arousal information is relatively preserved in old age and may interfere with goal-directed top-down information processing [69,70]. Future research needs to further differentiate the arousal level of emotional stimuli to investigate whether there is a cognitive control tradeoff between youth and old age when processing emotional information with different arousal levels. Second, the present study used emotional faces, which have the most social emotional significance, as stimulus materials, while people in real life rely more on contextual information for emotional information processing. Future studies need to consider other types of emotional materials and the influence of context on emotional information processing to improve the ecological validity of the study. Third, both proactive control and reactive control can activate the core brain region related to cognitive control—the prefrontal cortex (especially the lateral prefrontal cortex), and there are differences in activation modes between the two [22,23,37]. Individuals with a preference for proactive control have negative activation in the prefrontal cortex during the cue phase; individuals with a preference for reactive control have negative activation in the prefrontal cortex during the detection stimulus phase [33]. Future studies need to combine ERP, brain imaging, and fNIRS to monitor the activation patterns of the prefrontal cortex during the emotional AX-CPT task and to further reveal the underlying mechanisms from the neurophysiological level.

In addition, this study found differences in cognitive control tradeoff in emotional information processing between youth and older adults, thus suggesting that attentional processing of emotional information may be a resilient factor in maintaining well-being in older adults. Future research is still needed to further explore the positive and therapeutic qualities of attention to promote healthy aging in older adults.

## 6. Conclusions

In the present experimental conditions, the following conclusions were drawn.

(1) In emotional information processing, youth are more inclined to proactive control than older adults.
(2) In negative information processing, older adults are more inclined to reactive control strategies than youth; in positive information processing, older adults show similar cognitive control patterns to youth, and proactive control is enhanced.

## Figures and Tables

**Table 1:** Subjects’ demographic characteristics and cognitive performance.

	Young Adults (*n* = 30)		Older Adults (*n* = 26)		
	*M*	*SD*	*M*	*SD*	*t*
Age	19.53	0.94	69.65	2.83	−86.37 ***
F/M(ratio)	18/12		11/15		
Years of formal Education	13.70	0.65	14.58	1.58	−2.64 *
FTP	38.43	4.72	25.92	5.92	8.80 ***
PANAS-PA	33.50	4.42	33.96	6.01	−0.32
PANAS-NA	11.80	1.92	11.46	1.68	0.70
DASS-Dep	0.10	0.31	0.15	0.46	−0.52
DASS-Anx	0.23	0.57	0.27	0.53	−0.24
DASS-Strs	0.93	0.87	0.54	0.81	1.75
MoCA	26.87	1.41	25.65	2.56	2.15 *

Note. *M* = mean; *SD* = standard deviation; F/M(ratio) = female/male ration; FTP = Future Time Perspective Scale; PANAS-PA/NA = Positive Affect/Negative Affect scores on the Positive and Negative Affect Schedule (PANAS); DASS-Dep/Anx/Strs = Depression, Anxiety, and Stress scores on the DASS-21; MoCA = The Montreal Cognitive Assessment. * *p* < 0.05; *** *p* < 0.001.

**Table 2:** Four emotional AX-CPT sequence types design.

Cue Stimulus	Probe Stimulus	Percentage of Trials	Response
Happy (or sad)	Happy (or sad)	70%	Target response
Happy (or sad)	Neutral	10%	Non-target response
Neutral	Happy (or sad)	10%	
Neutral	Neutral	10%	

**Table 3:** Emotional face material rating results.

	Young Adults			Older Adults		
	Happy	Neutral	Sad	Happy	Neutral	Sad
Identity (%)	98.33	92.08	94.58	99.58	94.58	96.67
Arousal	6.83 ± 0.86	3.95 ± 0.60	6.62 ± 0.86	6.72 ± 0.75	3.88 ± 0.66	6.47 ± 0.81

## Data Availability

The final data and analysis files (in SPSS) could be retrieved from https://osf.io/6j9a5/?view_only=214932044c17421cb1b6ba6e35f20ac6 (accessed on 25 July 2022).
